# A longitudinal analysis of collapsibility with predictions over the southeastern Loess Plateau in China

**DOI:** 10.1038/s41598-021-02623-2

**Published:** 2021-12-10

**Authors:** Ziyu Zheng, Xi-an Li, Li Wang

**Affiliations:** College of Geology Engineering and Geomatics, Chang’an University, Xi’an, 710064 Shaanxi China

**Keywords:** Structural geology, Geomorphology, Sedimentology

## Abstract

Loess presents very unique collapsible behaviour due to its special under-compactness, weak cementation and porousness. Many environmental issues and geological hazards including subgrade subsidences, slope collapses or failures, building cracking and so on are directly caused by the collapsible deformation of loess. Such collapsible behaviour may also severe accidents due to sinkholes, underground caves or loess gullies. Moreover, with the increasing demand of construction and development in the loess areas, an in-depth research towards effective evaluation of loess collapsibility is urged. Currently no studies have made attempts to explore a rather complete and representative area of Loess Plateau. This paper thus provides a novel approach on spatial modelling over Jin-Shan Loess Plateau as an extension to experimental studies. The in-lab experiment results have shown that shown that the porosity ratio and collapsibility follow a Gaussian distribution and a Gamma distribution respectively for both sampling areas: Yan’an and Lv Liang. This establishes the prior intuition towards spatial modelling which provides insights of potential influential factors on loess collapsibility and further sets a potential direction of the loess studies by considering an extra dimension of spatial correlation. Such modelling allows robust predictions taken into account of longitudinal information as well as structural parameters and basic physical properties. Water contents, dry densities, pressure levels and elevations of samples are determined to be statistically significant factors which affect the loess collapsibility. All regions in Lv Liang area are at risk of high collapsibility with average around 0.03, out of which roughly a third of them are predicted to be at high risk. Clear spatial patterns of higher expected collapsibility in the southwest comparing to the northeast are shown adjusting for influential covariates. On reference guidelines for potential policy makings, county-level regions with the highest expected loess collapsibility are also identified.

## Introduction

**Figure 1:**
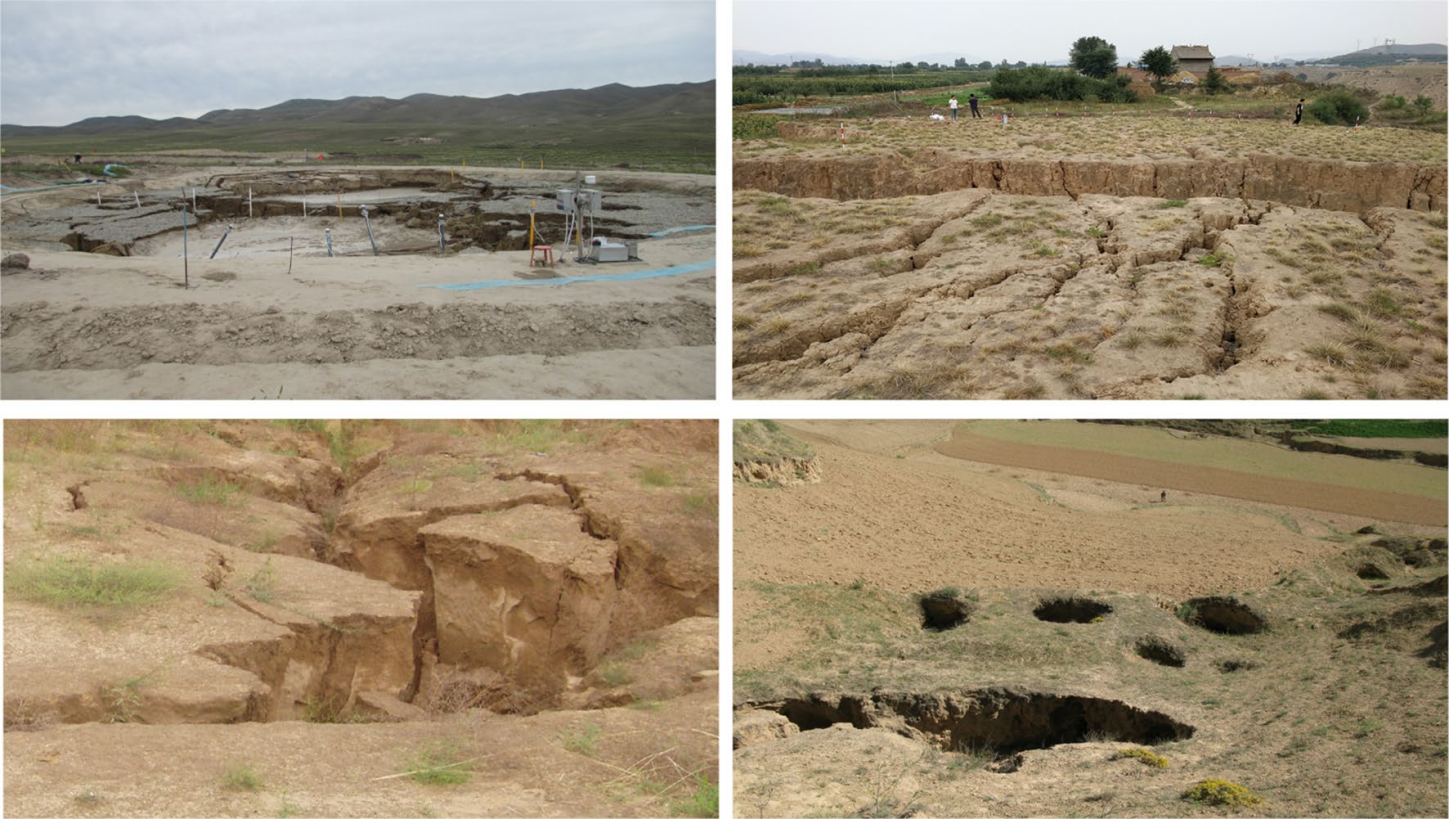
Demonstration of typical hazards caused by Loess collapsiility.

Loess is widely distributed across the world with the largest proportion sits in China which accounts for $10\%$ of the Earth’s land surface^1–3^. The Loess Plateau and Loess Plain together occupy approximately $7\%$ of the land territory of China^4,5^. Such huge coverage means that it is an unavoidable area of development within China in both human living activities and civil engineering construction programmes such as the ‘National Expressway Network Plan’ and ‘Mid-to-Long Term Railway Network Plan’^6^. As a kind of under-compacted, weak cemented and porous sediments, the extremely unique collapsibility of loess has remained under attraction over decades. The collapsible deformation of loess is often discontinuous and irreversible leading to extremely harmful consequences in all-round aspects covering construction, agriculture and transportation, to name a few. Typical geotechnical ones include the foundation collapse and slope stability. On a larger scale, the many environmental issues and geological hazards are likely to occur as a result of collapsible deformation of loess^1,7–9^. The erosional landforms in loess, such as loess pillar, loess walls, loess towers and loess caves compared with the topography of karst areas are also known as loess karst^10^. It is one of the typical environmental issues caused by collapsibility directly leading to to subgrade subsidences, slope collapses or failures, building cracking and so on. It may cause severe accidents in constructions associated with casualty and economic loss as the consequence of loess sinkholes or underground caves which are generally hard to detect. The formation process of loess gullies is also directly related to collapsibility where in low depressions, gullies are formed under water erosion. During such process, if gullies are steep, torrents and mudslides are likely to occur under water erosion. Such flooding is normally short in time but very sudden especially during times when storms are frequent. Figure 1 demonstrates some of the geological hazards caused by loess collapsibility. Such feature of sudden failures of loess collapsibility as well as the high potentials of hazards associated urge the investigation in effective and robust evaluation of soil collapsibility. With the increasing demand of constructions across the whole loess areas, spatial analyses over different loess types across a vast region are extremely informative. Based on the establishment of high risk factors to loess collapsibility and necessary location information, being able to predict the collapsibility across the Loess Plateau becomes equally important at the same time.

There are many researches delivering methods on evaluation of loess collapsibility which are mainly categorised into experimental and statistical methods. The most commonly applied experiment nowadays is the indoor immersion compression test which is comparatively less consuming in both time and finance. The fundamental method is based on the confined compression experiment which evaluates the site and field collapsibility based on the collapsibility index obtained. Many studies have made extensions to uniaxial or triaxial compression test^11–14^ as such method sometimes fails to represent the collapsible characteristics of loess under the natural stress level. Other indoor experimental methods include more complex extension which consists of more complicated multi-direction stress combinations and evaluation to collapsibility of humidified or anisotropic loess^15–18^. All of these aim to improve the accuracy of loess collapsibility evaluation despite still being affected by unavoidable disturbance during sampling, for example.

The other approach to evaluation of loess collapsibility is based on statistical modelling with establishment of relationship between collapsibility and some basic physical indices. Such method simplifies the experiments which are then used to induce the collapsibility coefficient based on the empirical relationship set. The relevant indices commonly used are the density and water contents which are reliably obtained with mature experimental methods. Table 1 presents some models proposed in previous studies where the influential factors mainly including water contents, porosity ratios and densities.

**Table 1:** Some models from previous studies.

References	Models	Covariates
^19^	$\delta _s = K(n - 40)(30 - w_n)$	$K = 0.05$ for loess; $K = 0.08$ for clayey loess; *n*: porosity; $w_n$: natural water content
^20^	$\delta _s = 0.1283 - 0.4014(w/e)$	*e*: porosity ratio; *w* water content
^21^	$CP = 45.506 - 0.072(S - C)$ $- 0.439w_0 - 3.123\gamma _d + 2.851\ln (P_w)$	CP: potential of collapse; $w_0$: initial water contents; $\gamma _d$: dry bulk density; *S*: sand contents; *C*: clay contents; $P_w$: post water press
^22^	$\delta _s = 0.015 + B(\gamma _i - \gamma )$	$\gamma$: wet density; $\gamma$: initial wet density; *B*: slope of regression
^23^	$\delta _s = A\exp \{BJ\}$	*A*, *B*: parameters to be estimated; J: ratio of large-medium sized pores
^24^	$\delta _s = 0.0345 + 0.00423e_0$ $+ 0.0161\exp \{-(r - 18)^2/100\}$ $+ 0.0134\exp \{1.7w\}$	$e_0$: porosity ratio; r: clay contents w: water contents.
^25^	$\delta _s = a \exp \{b/S_r\}$ or $\delta _s = a + bK$	*a*, *b*: parameters to be estimated $S_r$: saturation; *K*: compaction.
^26^	$\delta _s = -0.8187C + 0.4618$	*C*: compaction ratio at the most optimal water contents.
^27^	$\delta _s = 0.013w - 0.022\rho _d + 0.366e$ $-0.017we + 0.020\rho _de - 0.265$	*w*: water contents; $\rho _d$: dry density; *e*: porosity ratio
^28^	$\delta _s = -0.00261w - 0.53863\rho _d - 0.28864e$ $-5.56939 \times 10^{-4}E_s + 1.12522$	*w*: water contents; $\rho _d$: dry density; *e*: porosity ratio; $E_s$: compression modulus.
^29^	$\delta _s = 0.0295 - 0.0027w + 0.0154\exp \{1.7e\}$ $-0.0286 \exp \{-\frac{(c - 18)^2}{100}\}$.	*w*: water contents; *e*: porosity ratio; *c*: clay contents

One of the well established determinants causing loess collapses is its structure^30–32^. In simplified terms, the loess structural failure occurs as the pore structures gradually collapse under water or other external forces such as tectonic uplift or earthquakes^9,33^. Such deformation is related to a combination of factors representing the structural parameters, basic fabric properties and spatial environmental features. The microstructural features act as the dominating factor which can be characterised under pore structures^33–35^. Pore structures are shown to affect the water retention^36^ and appear to be rather unstable when loaded with pressure or watered^37^. Similarly to the models listed in Table 1, the initial water contents and dry densities within the category of basic fabric properties are both shown to affect the collapsibility coefficient adversely^14,38–40^. On the macroscopic, it is intuitive that the environmental features such as precipitation and vegetation coverage certainly affects the internal feature of the loess. These could be reflected on the locations of sampling; the closer the locations are the more similar environmental conditions the loess samples are under.

Note that even though the current models vary in covariates, all of them are still limited to the inclusion of only basic physical properties. There are optional ones estimating the collapsibility via static sounding, pressuremeter test and resistivity^41–43^. Despite all proposed models, the modelling methods are mostly based on variations of simple linear regression. None of the studies had made attempts towards a systematic research over the large area of Loess Plateau. This means that the vast amount of spatial information contained within the loess distribution has not yet been utilised. Often in reality, longitudinal data allow one to evaluate differences across regions, distinguish spatial clusters from random noises and identify potential exposures of high risk areas. Geostatistical methods^44–47^ are commonly employed to model such data and establish potential spatial relationship among regions. The extensive applications of this type of models have show satisfying results in fields including geology and environmental science. Such spatial modellings are however rarely seen in areas of loess studies not to mention the collapsible behaviours of loess. Despite previous researches^39,48–50^ have briefly compared the different behaviours of collapsibility between areas and soils, no sophisticated mathematical model has been adapted to describe such observations. The detailed analyses over a smoothed layer representing whole distribution of a Loess Plateau are never investigated.

In this paper, a Gaussian generalised additive model^51^ with both fixed and random effects is employed to fill in the blank area of spatial modelling in loess collapsibility. Such model utilises the coordinate information consist within the data and fully describes the spatial variation existing given the fixed effects being taken care of. This allows the mapping of collapsibility of soil at regional levels as well as predicted location spots given coordinates. Such ability to analyse regional collapsibility trends, taking into account of a combination of structural parameters, basic physical properties and location information, is extremely useful in understanding the collapsibility feature of loess at a bigger scope. The exploratory study is carried out based on in-lab experiments where the potential influential factors of collapsibility features are identified with collapsibility trends captured using simple multiple linear regressions. The models are justified via residual checks and cross-validated by comparing the predicted and observed values. In this article, we seek to address the following research questions: (1) Under lab-conditions, what are the potential risk factors affecting loess collapsibility? (2) How does collapsibility trends vary across Lv Liang area with given location coordinates? (3) Aggregating to county levels, what are the areas with highest risk of loess collapse compared to the regional average? (4) What is the nature of the relationship between collapsibility and lab-identified factors?

## Results

### Exploratory results

The initiation for exploratory study considered the behaviour of collapsibility from a combination of effects of dry density, water content, porosity ratio and press. The log-transformed collapsibility obtained from the indoor experiment on Yan’an samples is estimated to have the following form:

$$\begin{aligned} \log (\text {collapsibility}) =&10.21 - 16.98 \times \text {water contents} -4.79 \times 10^{-4} \times \text {press} \\&- 2.81 \times \text {porosity ratio} - 6.26 \times \text {dry density}. \end{aligned}$$

The estimated parameters in the multiple linear model regarding are given in Table 2. All covariates appeared to be statistically significant in explanation of collapsibility based on experimental results.

**Table 2:** Estimated covariate coefficients for multiple linear models.

	Estimate	Std. Error	t value	Pr($>|t|$)
(Intercept)	10.21	0.67	15.23	$<10^{-5}$
Water contents	$-$ 16.98	1.53	$-$ 11.10	$<10^{-5}$
Press	$-4.79 \times 10^{-4}$	$1.14 \times 10^{-4 }$	$-$ 4.21	$<10^{-5}$
Porosity ratio	$-$ 2.81	0.59	$-$ 4.74	$<10^{-5}$
Dry density	$-$ 6.26	0.27	$-$ 22.82	$<10^{-5}$

All covariates are negatively associated with collapsibility. That is saying that the higher water contents, press levels, porosity ratios and dry densities are, the lower collapsibility coefficients are. The collapsibility appears to be most sensitive to water contents, i.e. one unit change in water contents result in 16.98 units change in collapsibility. One thing to note that the negative impact of porosity ratio is due to the fact that they are calculated for each different pressure here. It is possible that the higher the pressure, the more compacted particles are which leads to an increase in the specific gravity of solid particles. This could further cause such negative relationship presented in data.

**Figure 2:**
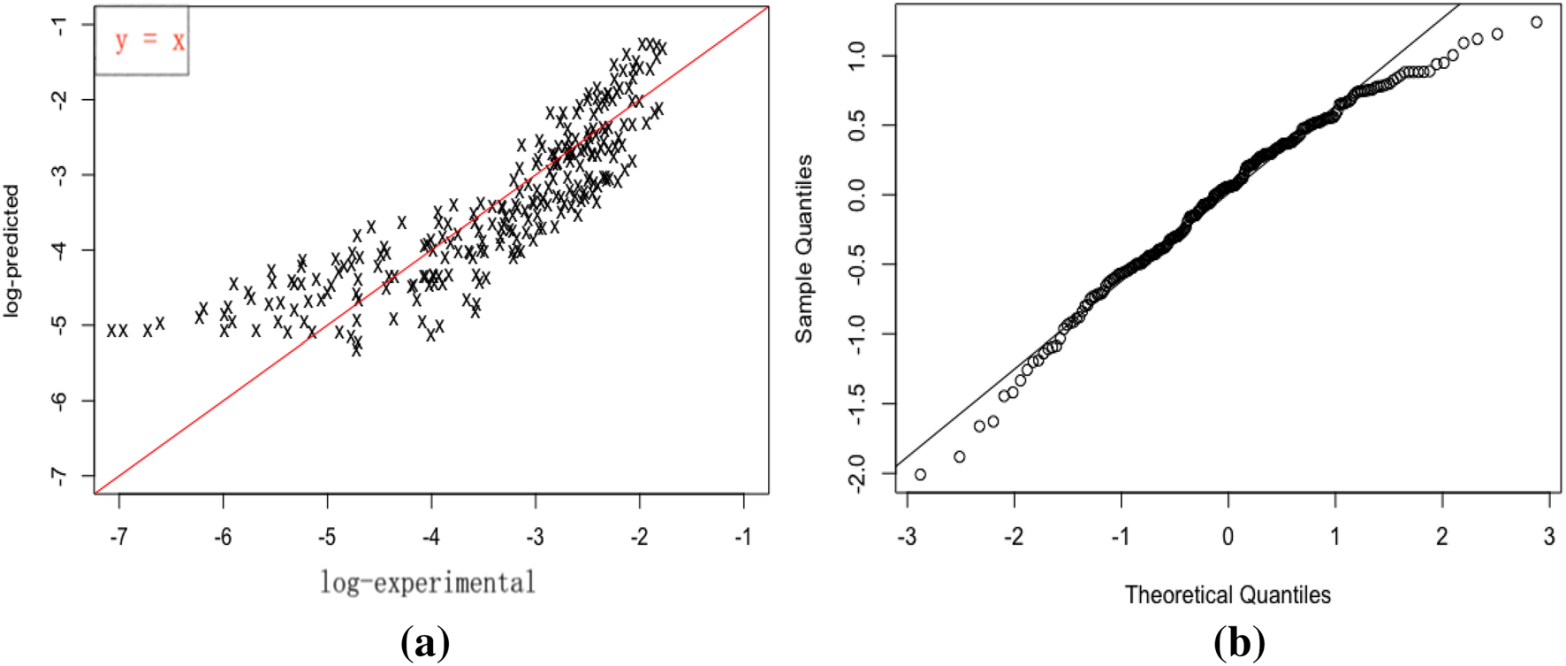
Multiple linear model validation: samples from Yan’an; (**a**) Predicted vs experiment (log), (**b**) QQ-plot for residual checks.

The $R^2$ value being 0.74 suggests that the model explains $74\%$ variation contained in the data. The model predictions are validated against the experimental results as shown in Fig. 2a. The plot demonstrates that at log-scale, most of the data fall on the $y = x$ reference line with the left tail being slightly off. This means that most of the data are well described and accurately predicted based on the multiple linear regression model as an exploration (supporting $R^2$ value). The variation of predicted against experiments also appears rather even across the whole range, which suggests that the model provides rather robust predictions. The majority of model residuals fall roughly along the one-to-one reference line as required in the assumption (Fig. 2b). The slightly off two tails are potentially affected by the positive skewness shown (see Fig. 4).

**Figure 3:**
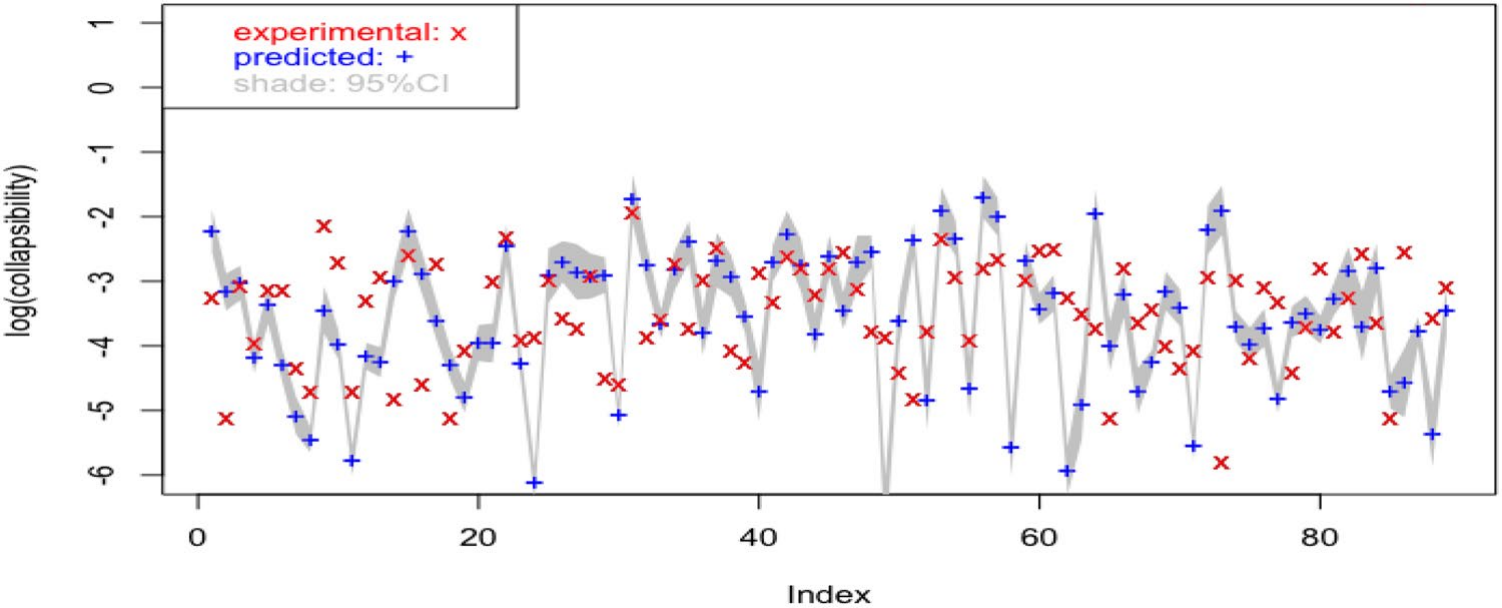
Predicted values with $95\%$ confidence interval overlaid by observed values at log-scale: multiple linear model validated on samples from Lv Liang.

The model is further validated against the loess samples taken from Lv Liang area. Figure 3 shows the predicted values based on multiple linear regression against the experiment results together with its $95\%$ confidence interval. It can be seen that the majority of experimental values fall within the predicted $95\%$ confidence interval, which suggests reasonably accurate predictions especially considering the loess samples are taken from a complete different area. The model is however, subjected to further improvement as allowance for uncertainty is relatively narrow (see gray shades in Fig. 3).

### Microstructure: pore connectivity

**Figure 4:**
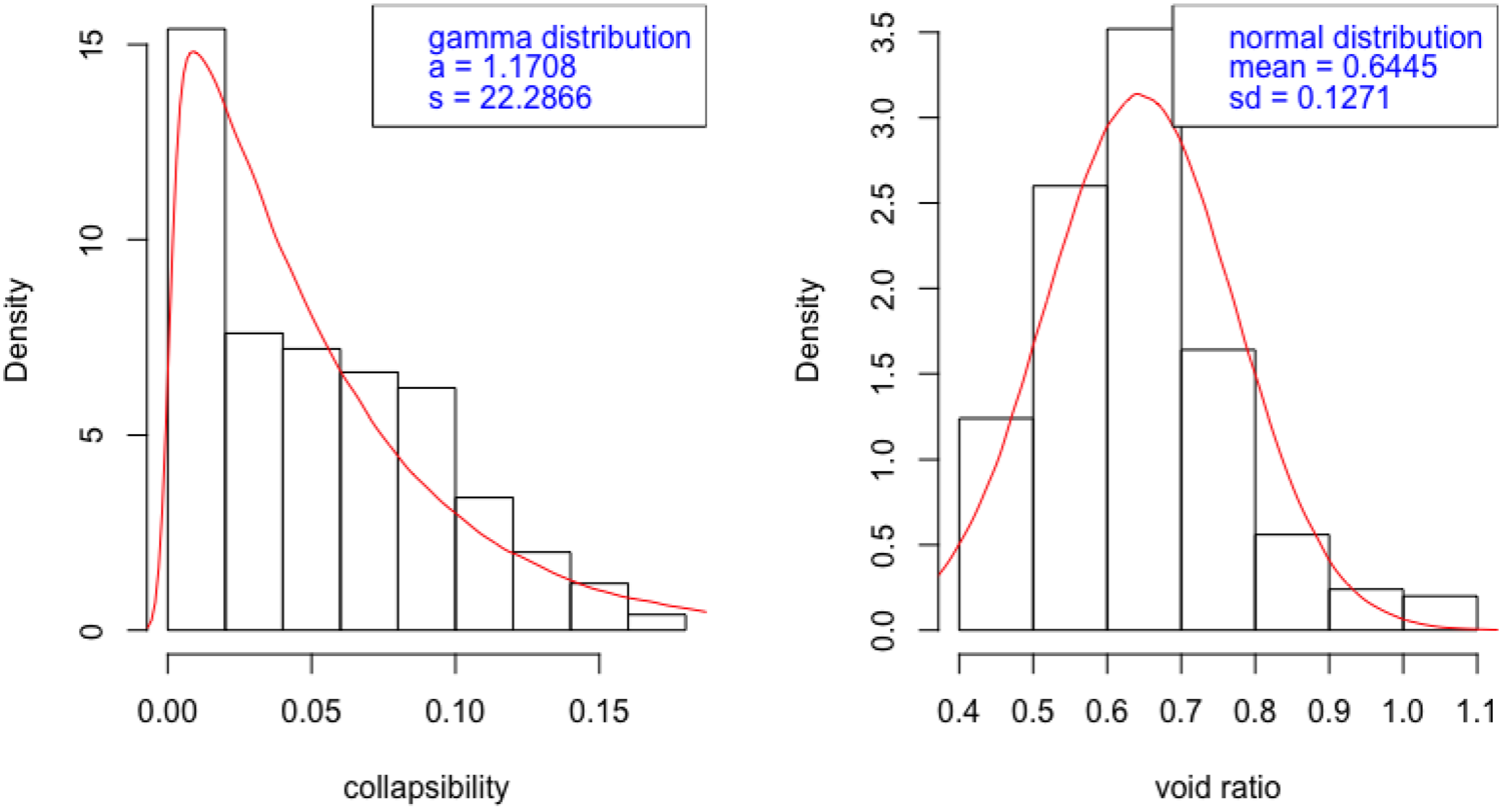
Probability description of collapsibility and porosity ratio: Yan’an Area.

**Figure 5:**
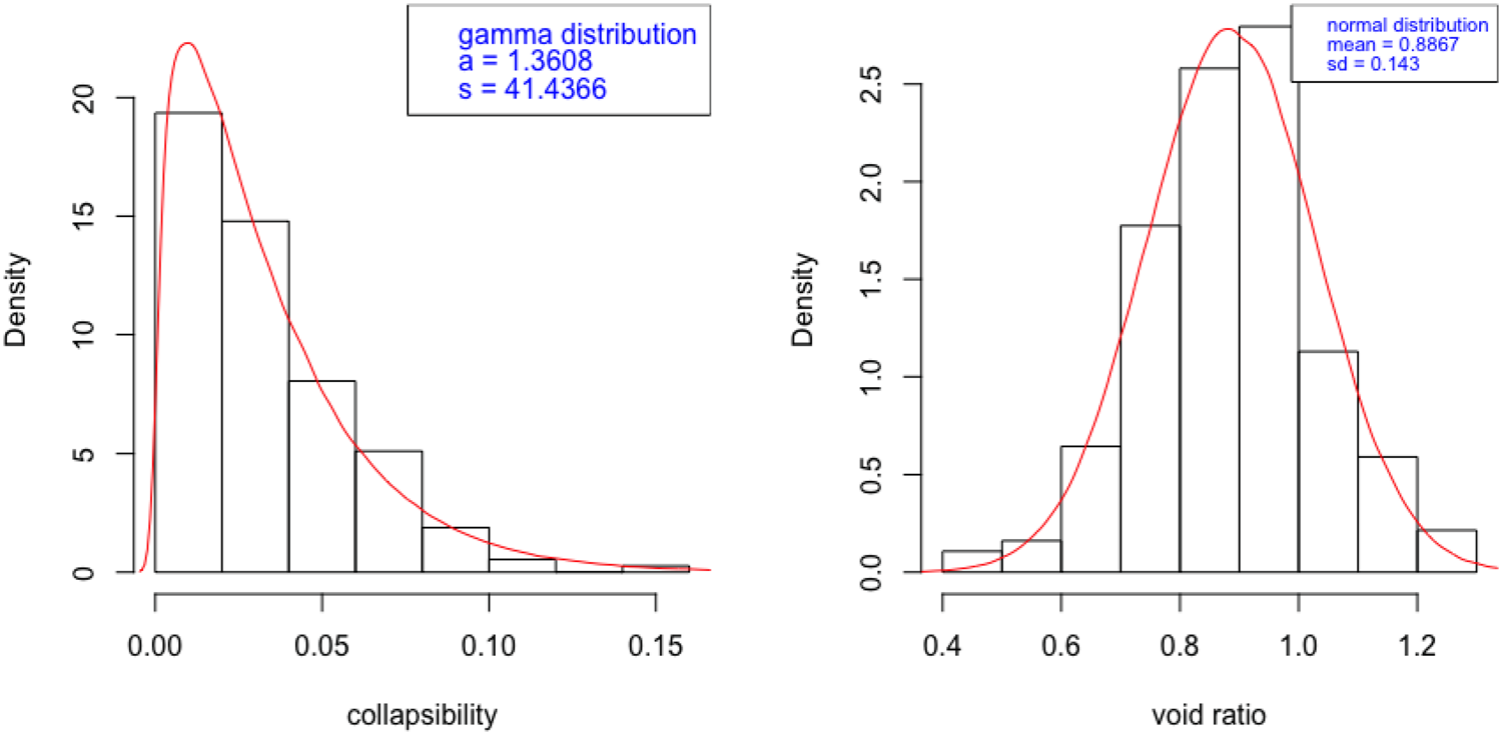
Probability description of collapsibility and porosity ratio: Lv Liang Area.

For both sets of samples used in this study, the collapsibility coefficient follow a Gamma distribution as Figs. 4 and  5 show that the distribution of collapsibility is skewed to the left. It follows a Gamma distribution with shape and scale parameters denoted by *a* and *s* whose probability density follows

$$\begin{aligned} f(x) = \frac{1}{s^a\Gamma (a)} x^{a - 1} \exp \{-\frac{x}{s}\}, \end{aligned}$$

where $x \ge 0$, $a > 0$ and $s > 0$. The similar manner of fitting a probability distribution is also done to describe porosity ratio; the distribution is rather symmetric and thus a Gaussian distribution fits nicely. The similar pattern means that the collapsibility behaviour and the composition of pore structure share the same probability characteristicsfor the two areas.

**Figure 6:**
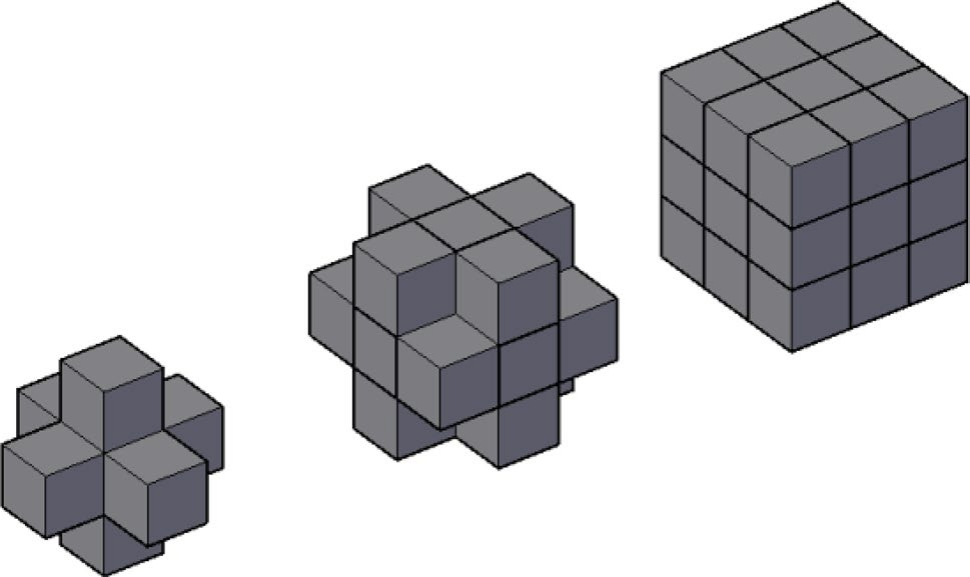
Demonstration of network.

As a kind of porous medium, there are a lot of pores and connections among them in loess. The complex links between pores and their connections forms a network structure. Considering the cubic shaped voxels, there are three common structural elements in three-dimensional connectivity detection operation: 6 adjacent (voxels with a common surface are regarded as connected), 18 adjacent (voxels with at least one common edge are regarded as connected) and 26 adjacent (voxels with at least one common vertex are regarded as connected), as shown in Fig. 6. This paper extends the 26 adjacent network to the connectivity of pores.

**Figure 7:**
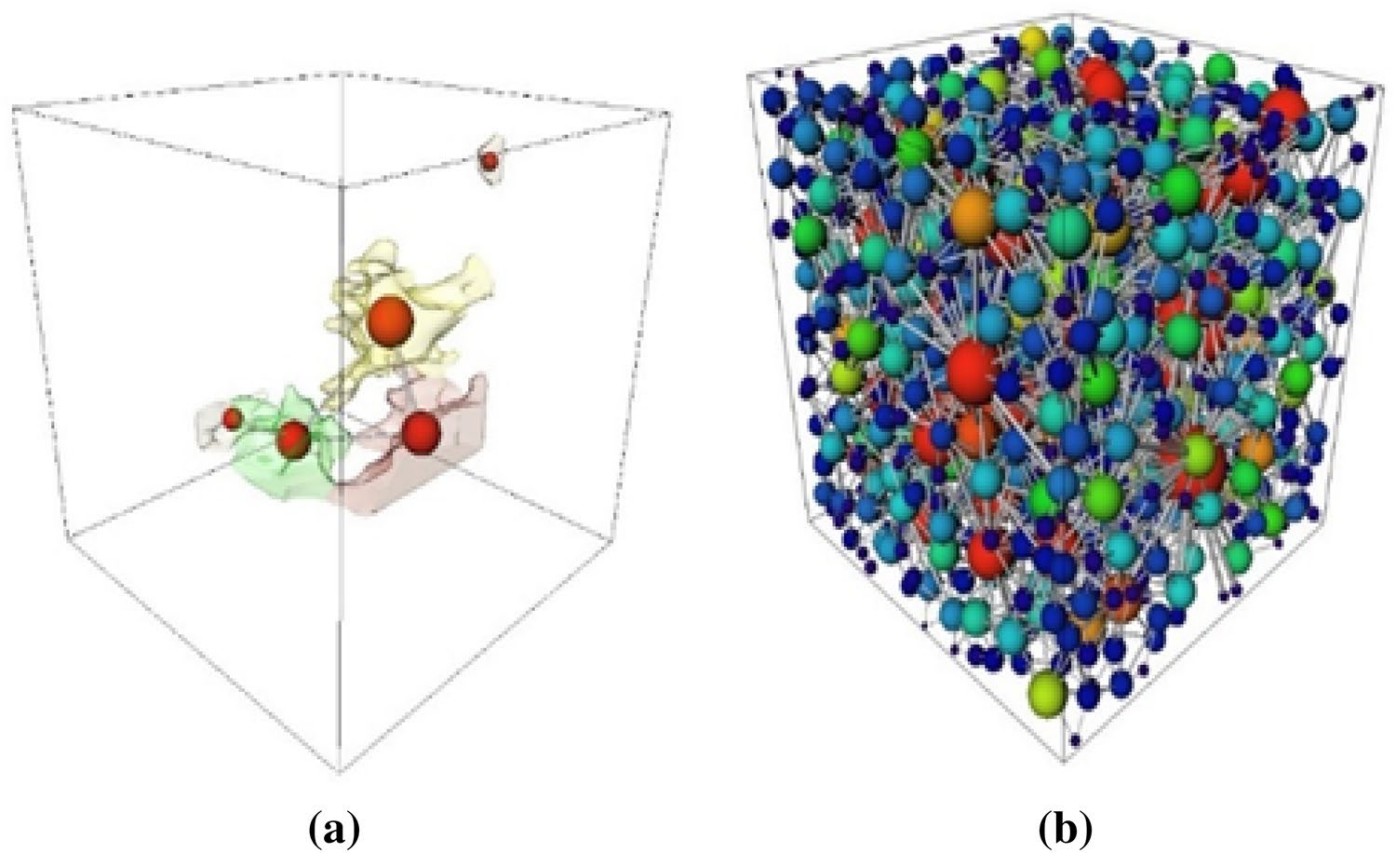
Demonstration of reconstructed pore network of one Loess sample from Lv Liang; (**a**) Abstracted pores (ball) and connections (stick), (**b**) pore network.

Thus it is intuitive to treat the connectivity of pores as a more relevant factor of collapsibility of porosity ratio. In the process of understanding and studying the connectivity of this network structure, the pore network model abstracts the porous medium as an ideal geometry (ball) and the throat as a hollow stick with variable length. This is based on the neglect of the pore volume, tortuosity of connections and other factors (Fig. 7a). Figure 7b shows a complete collection of pore network modeling.

**Figure 8:**
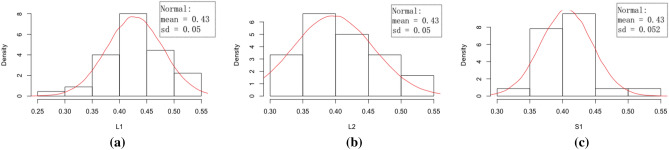
Pore network; (**a**) probability distribution of degree distribution for L1, (**b**) probability distribution of degree distribution for L2, (c) probability distribution of degree distribution for S1.

The quantification of pore network can be defined via degree distribution (k). It represents the number of sticks connected to the ball, that is, the number of connection paths in the network. In complex networks, the degree of a node represents the number of edges adjacent to the node. Such information is fully contained within the microstructural scans of loess samples. As the microstructural extraction based on image analyses is not of focus in this paper, the techniques are omitted here. Similar approach can be found in more details in Ref.^37^. Based on image analysis techniques, the porosity degree distribution satisfies a Normal distribution for each layer respectively (Fig. 8) with no clear difference presented. In the Lv Liang loess samples, only a small number of pores are joined by a large number of connection paths. Despite the small number, the more connected the pores are, the more likely they are to directly affect the collapsibility behaviour of loess. Considering both of these two measurements describes the behaviour of pores, the model extension further considers the connectivity of pores as one influential factor of collapsibility.

### Spatial distribution of Loess collapsibility via generalised additive model

**Figure 9:**
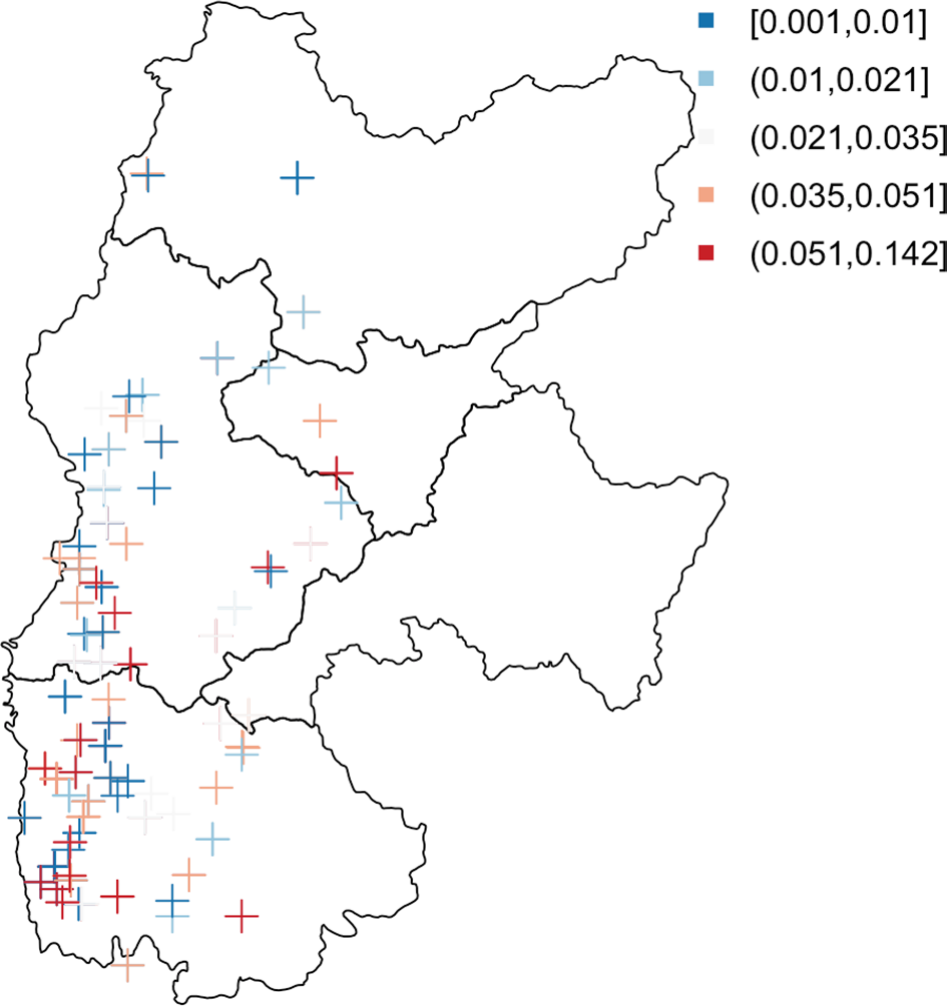
Map of collapsibility coefficients over Lv Liang area.

With the location information, the collapsibility coefficients for samples taken from different locations across Lv Liang area shows a fairly clear spatial pattern (Fig. 9), where similar colours tend to appear next to each other; the existence of spatial clustering means that there are potential underlying spatial random effects.

To evidence the observed spatial clustering in collapsibility, the Moran’s I measurement which is based on both feature locations and values simultaneously is used. It is commonly used to evaluate whether the patterns are clustered, dispersed or random. It is calculated as:

$$\begin{aligned} I = \frac{n}{S_0} \frac{\sum _{i = 1}^n\sum _{j = 1}^n w_{ij}z_iz_j}{\sum _{i = 1}^n z^2_i}. \end{aligned}$$

Here $z_i$ refers to the deviation of collapsibility at location *i* from the mean collapsibility. It is multiplied by the spatial weight $w_{ij}$ between collapsibility at locations *i* and *j*. For the calculation in this paper, the weights are negatively proportional to the distance between *i* and *j*. $S_0$ is the aggregate of all spatial weights, i.e. $\sum _{i = 1}^n\sum _{j = 1}^n w_{ij}$. The Moran’s I statistic calculated based on real data is then compared against *I* calculated based on data generated with complete spatial randomness. In this data set, the *p*-value being 0.04 implies that the spatial clustering exists at $5\%$ significance level.

**Figure 10:**
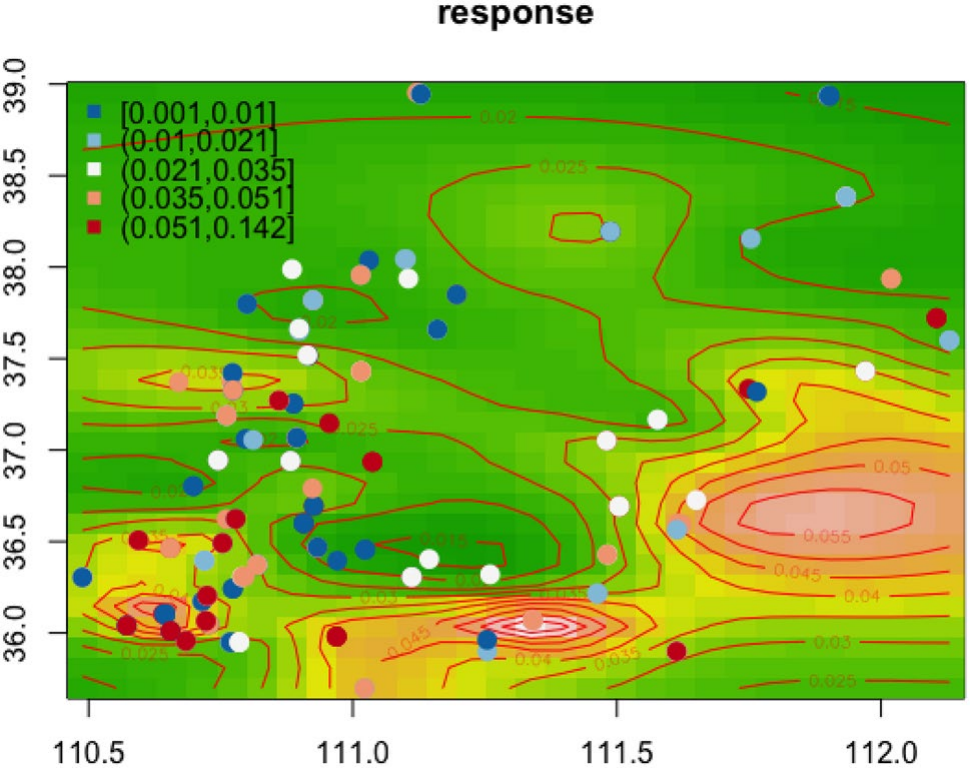
Spatial contours of predicted collapsibility contour with original values overlaid; x-axis: lontitude; y-axis: latitude.

**Figure 11:**
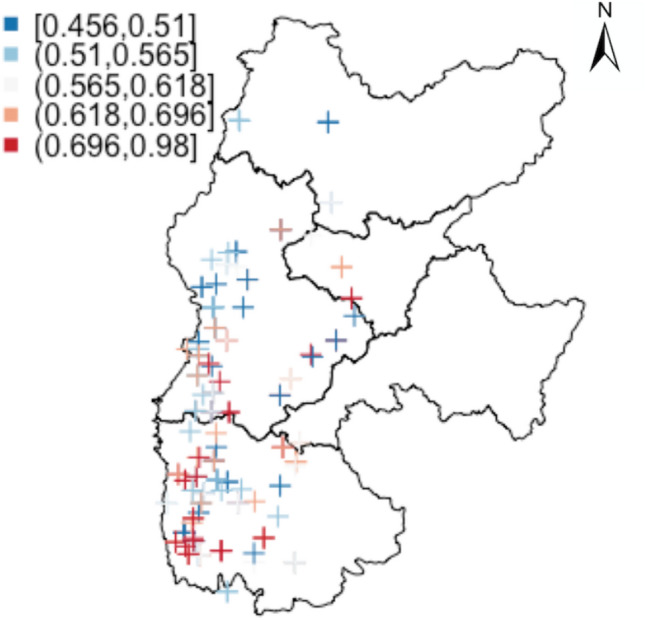
Probability of each sampling location with estimated collapsibility greater than 0.015.

The southern part of Lv Liang in the Jin-Shan Loess Plateau is predicted to preserve higher collpasibility coefficients under generalised additive model with smoothed spatial residuals. The estimated collapsibility coefficient values are mapped with longitude and latitude information given in Fig. 10. The higher collapsibility predictions are surrounded by the red coloured contour lines. This is justified by the sampling location with high observed collapsibility fall within the red-coloured contour circles. Figure 11 instead of presenting the predicted collapsibility values over the map, it shows that the relative risks as to how collapsible the soils are adjusting for known influential factors. The probability demonstration allows one to take care of uncertainties during interpretation. In general, probabilities can be thresholded to 5 different intervals: [0, 0.2], (0.2, 0.4], (0.4, 0.6], (0.6, 0.8] and (0.8, 1]. Each of these indicate low risk, mild risk, moderate risk, high risk and severe risk. The two Figures demonstrated rather similar spatial patterns where high collapsibility risks appear near the southern part of Lv Liang area.

**Figure 12:**
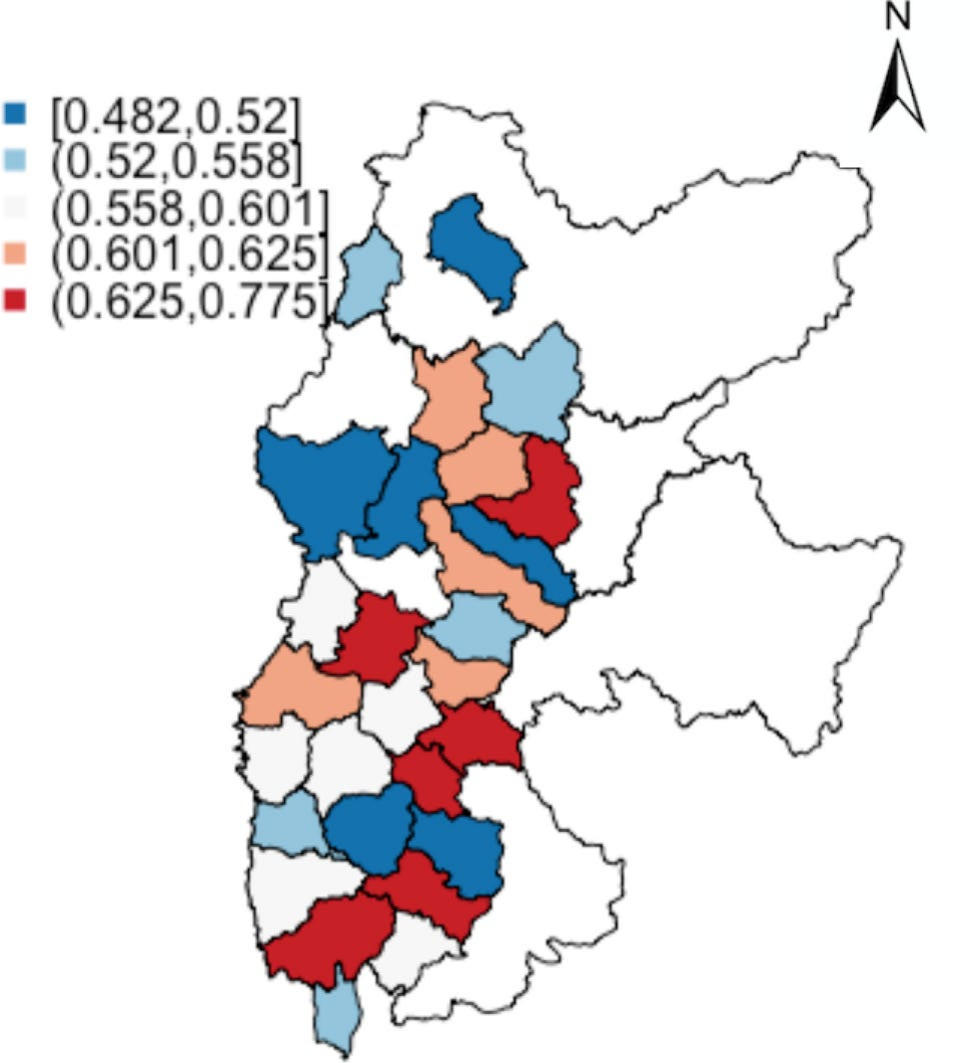
Lower administrative areas with high risk of collapsibility.

It is often helpful to present administrative-level results in terms of provide guidance to relevant authorities. There are in total 28 lower administrative regions (Xian) of which samples are taken from. Again, Fig. 12 maps the risk of each region having estimated collapsibility greater than 0.015. Table 3 shows the ones which are estimated to have severe and high risk of collapsibility being greater than 0.015 (i.e. ${\mathcal {P}}(\text {estimated collapsibility}> 0.015) > 0.6$).

**Table 3:** Table of lower administrative regions predicted to be at high risk of collapsibility.

Name	Zhongyang	Gujiao	Linfen	Xiangning	Fenxi	Lingshi
${\mathcal {P}}({\hat{y}} > 0.015)$	0.7746	0.7709	0.6834	0.6781	0.6623	0.6319
Name	Lan	Xiaoyi	Wenshui	Loufan	Shilou	Jiaokou
${\mathcal {P}}({\hat{y}} > 0.015)$	0.6153	0.6107	0.6098	0.6021	0.6020	0.6009

${\hat{y}}$ refers to the estimated collapsibility coefficient.

## Discussion

Under pre-determined dry densities, press levels and water contents, it has been found that for dry densities under 1.6, the collapsibility reaches peaks under 500 kpa press levels. The samples of 1.6 dry density tends to bear a lot higher press levels at about 1500 kpa. Such trends are shown in regardless of water contents within the sample. One conjecture can be made that for typical Malan loess samples, a sudden change is involved in the collapsibility behaviour when dry density reaches certain level. Upon model selection procedures, water contents, press levels, porosity ratios and dry density are all taking important roles as covariates. All of the four are statistically significant and have negative effects over collapsibility (refer to Table 2). Water content appears to have greatest effect size over log-transformed collapsibility followed by the dry density. This also agrees with the observations in Fig. 16. The cross-validation in this model supports the adequate covariate selection and evidences the impacts water contents, press level, porosity ratios and dry densities have over loess collapsibility in a quantitative manner. These influential factors are also intuitive on the environmental perspective. As the process of loess collapse is commonly caused by pipeline or groundwater infiltration. Thus water influences can be seen as the external causation of loess collapsibility. When sufficient pressure and appropriate humidity are not available at the same time, the loess layers are likely to be poorly compacted. This results in the loess layers which are relatively low in water contents but high in porosity which in turn leads to under consolidated loess. Thus loess collapsibility shows as a final product which highly correlates to porosity ratios in this study. These results all agree with previous studies presented by Refs.^33–35,37^, for example.

Despite the reasonable results under linear settings, spatial patterns are still presented as indicated by both the observations and the skewness in residuals of linear models. The generalised additive model allows inclusion of smoothed parameters. This is more intuitive as the factors identified above are all continuous in nature. The smoothed prediction surface presents the predictions of all possible location on surface of Jin-Shan Loess Plateau in Lv Liang area. The highest estimates fall in Southern parts, especially towards the Southeast of the study area. The presentation in terms of probability of likelihood to experience collapsibility at locations suggest that there’s a higher chance for soils in southwestern of the study area to be more collapsible. This coincidences with the distribution of sandy belt, silty belt and clayey belt of loess; it shall be expected that the more southern it is, the more clayey the loess are. Such regional distribution also agrees with findings in Refs.^52,53^.

In terms of aggregated areal level study, the regional average of Lv Liang (around 0.03) is a lot greater than the threshold of collapsible soil (0.015); the case holds true when disaggregated to sub-administrative areas at county-level. More southern regions presented higher risks comparing to the regional average, indicating higher risks of suffering hazards due to collapsible loess. In particular, the top ten regions with high risks to severe risks are listed in Table 3. This table is useful as a guideline to authorities when making relevant construction decisions or environmental policies.

One of the drawbacks of this paper is the incompleteness of data over the whole Loess Plateau. This is to say that interpolations are made on the smoothed surface when making predictions. At the same time, despite that the model is easily extensible to other loess areas, the limited location information may result in a not so general application. Further research aims to see a more complete sample representing the Jin-Shan Loess Plateau. This allows a more detailed risk map at the lower administrative regions in practical guidance. More data over other ecological covariates such as rainfall which represents the temporal trends would certainly build a bigger general picture of loess collapsible behaviours among the plateau and draw links to other potential relevant geological hazards.

The study consists of two main areas including a lab-based experiment condition modelling and a full distributional data modelling else where. It demonstrates an effective combination of the two through a simple multiple linear regression model in exploratory analysis and an extension to longitudinal data which takes care of the spatial random effects. Such approach is not seen in the research area of loess collapsibility studies. The experiments demonstrated the distinct effects of dry densities and water contents on collapsibility which are then supported statistically through relevant models. Satisfactory results are shown via residual checks and the cross-validation process. The distribution of grain size distribution certainly is an interesting aspect when talking about collapsibility. However, it is not of the main interest in this study in particular. The study of pore structure in “Discussion” to certain extent reflects the grain sizes where the pore structure is deemed to be a statistically significant factor to the collapsibility behaviour. This result is based on the Yan’an sample and validated against the Lv Liang Sample (Fig. 8).

This paper introduced a novel approach on how influential factors at structural, basic physical properties and environmental level affect collapsibility of loess. Based on findings in general experimental studies, an extension of generalised additive model is adapted to consider the existing spatial random effects preserved within the data. The paper successfully presented a cross-validated prediction map of soil collapsibility over Lv Liang area, with covariates fully justified and selected in the experimental linear model. A clear pattern of high collapsibility in the Southwest comparing to that in the Northeast is evidenced statistically based on data. It presents how distributional tendency is in terms of loess collapsibility and the relevance to each of the covariate of interest at the same time. At aggregated county level, the highest risk areas are identified which can be potentially useful for decision making. Extensions towards both spatial and temporal trends are easily available given appropriate data.

The establishment of influential factors over loess collapsibility is of great importance when describing how each covariate affect the loess collapsibility both in size and magnitude. Thus when considering the environmental impact relevant to loess collapsibility, certain policies making alterations to these factors can be introduced correspondingly. Combined with the spatial modelling and predictions, it is of environmental significance to provide loess collapsibility information across the regions in order to make alerts to hazards such as karsts, gullies, landslides and mudslides mentioned above.

Further studies on both longitudinal and regional-level data combining with other relevant ecological data including rainfalls or vegetations over the plateau, for example, should also be done to be able to draw firm conclusions over how the collapsibility behaves and evolve over times in the sense of environment. The general picture of linking to potential environmental hazards can be provided based on forecast models when relevant data are available.

## Methods

### Study area

**Figure 13:**
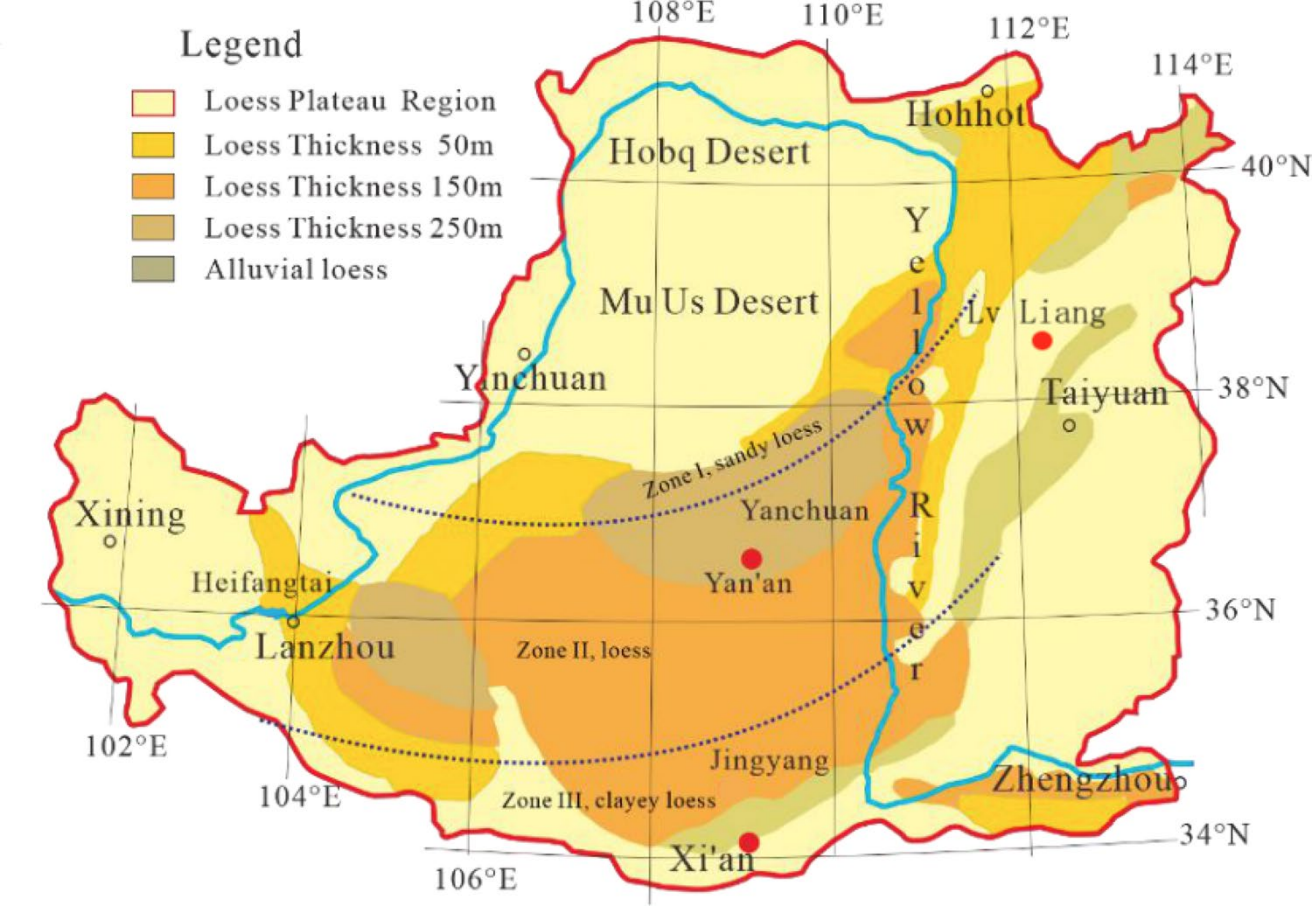
Cities of sampling marked on the Loess Plateau with Loess Belts.

**Figure 14:**
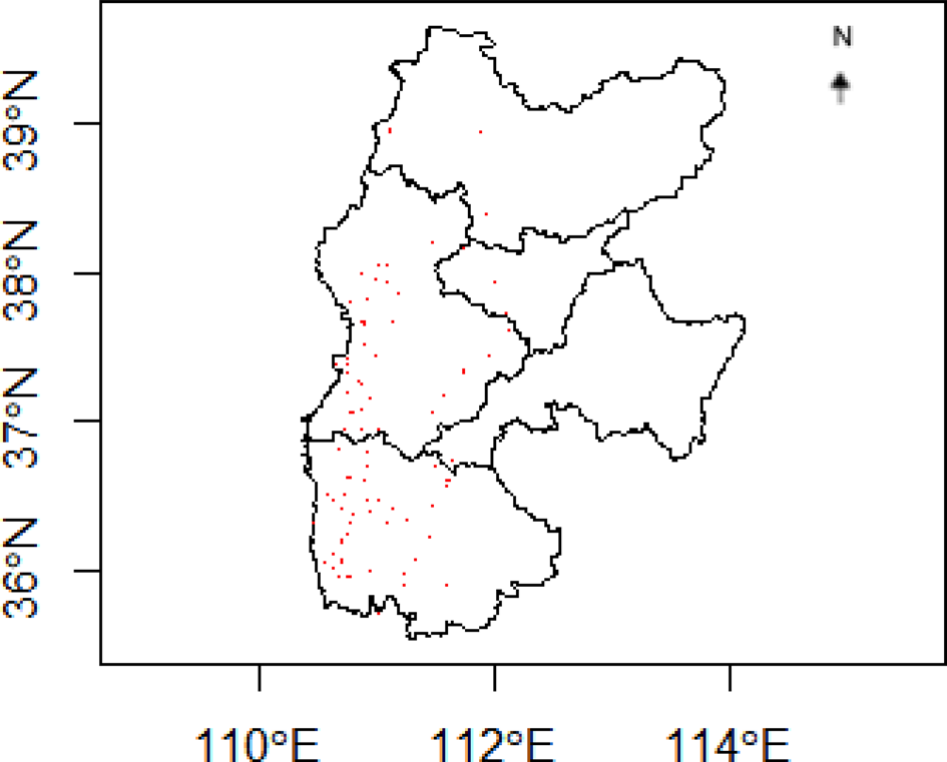
Lv Liang sampling locations mapped on Jin-Shan Loess Plateau (red dots); sketch via R.

Two sets of samples are used in this study. The samples for the exploratory study based on indoor experiment are the late Pleistocene loess (Q3 Malan loess) taken from Yan’an city, Shaanxi Province at the 4-m depth. It is from the northern section of construction site of Qingliang Mountain (N36$^\circ$ 39’, E109$^\circ$ 29’). On extension to the spatial modelling towards the loess collapsibilit y, the samples are from Lv Liang, Shanxi Province. There are in total 191 samples from 80 different locations with different depths and elevation used as a complete representation of Jin-Shan Loess Plateau. Figure 13 marks the two cities from which samples are taken, where the detailed locations in Lv Liang are shown in Fig. 14.

### Sampling methods

The original samples are carefully taken out and cut into small and uniform blocks with as few calcareous nodules as possible. They are dried in a cool and ventilated place for 10 days. The basic physical properties such as water contents and dry densities are obtained using oven-drying and cutting ring methods respectively according to the Specification of Soil Test (SL237-1999)^54^. The specific gravity of soil particles are measured via pycnometer method. Porosity ratios are calculated based on dry densities via $e = \frac{\rho _s}{\rho _d} - 1$, where $rho_s$ can be induced given specific gravity of solid particles. The collapsibility coefficients are obtained via the double oedometer method^55^. The coefficients of collapsibility are calculated for each sample with formula: $\delta _s = \frac{h - h'}{h_0},$ where *h* and $h'$ refer to the stablised height and under given pressure and the height of stablised sample after adding water. $h_0$ refers to the initial height before any pressure is applied. The collapse potential of loess are often categorised into the following intervals: (0, 0.015], (0.015, 0.03], (0.03, 0.07] and (0.07, 1]. Each of these indicates non-collapsible, weakly collapsible, moderately collapsible and strongly collapsible respectively according to Ref.^56^. The in-lab experiments are done based on the samples from Yan’an and a summary of the basic physical properties and collapsibility indices for all samples is given in Table 4.

**Table 4:** Summary of coefficient of collapsibility and other basic physical properties for Loess samples; Yan’an.

	Collapsibility	Water contents	Porosity ratio
Min.	0.00085	0.12	0.4140
Q1	0.01702	0.14	0.5488
Median	0.04430	0.16	0.6400
Mean	0.05253	0.16	0.6445
Q3.	0.08228	0.18	0.7075
Max.	0.16850	0.20	1.0619

*Min* minimum, *Q1* 1st quantile, *Q3* 3rd quantile, *Max* maximum.

**Figure 15:**
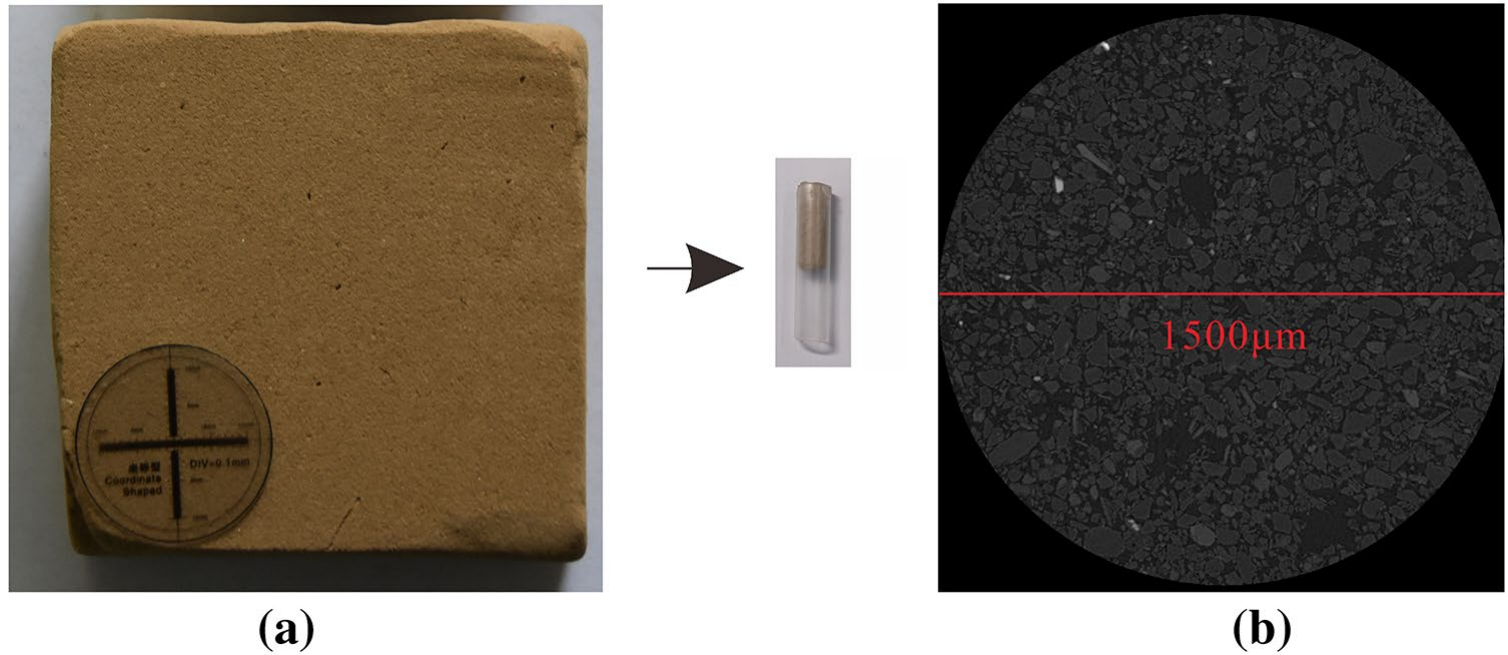
Demonstration of prepared sample and scanned outcome; (**a**) dried and tubed sample, (**b**) scanned sectional image.

For scanning purpose, the Lv Liang samples are further processed. The selected dried soils are then cut into cylinders with base diametre of 0.5 cm and height 1 cm. Such small-sized and regular-shaped samples are made for accuracy and time efficiency. These cylindrical samples are then preserved in hard transparent plastic tubes for scanning. The tubes are sealed with superglue and films to avoid transportation during scanning process. Sectional scans are obtained by ZEISS Xradia 520VersaCT machine with horizontal resolution 1.5 $\upmu m$ and vertical resolution 4.5 $\upmu m$. Samples with representative areas of $1500 \times 1500 \times 450$ $\upmu m$ are scanned and each cylindrical sample has 101 continuous gray-level sectional images as an outcome. Figure 15 shows an example of the dried and tubed sample as well as a sectional image outcome from $\upmu$CT scan.

The samples are taken from the Lv Liang regions where the secondary scanning samples are mainly concentrated on the Loess of layer L1 as L1 loess occupies a dominant position. To allow more freedom in soil types, the model also relaxes the restrictions to allow few collections from L2 and different layers of paleosol (S1, S2, S3 and S5) other than layers of loess. The rest of the layers are very rare and thus they are grouped into one category as ‘others’. These are further distinguished in the modelling part as a factorised effect. Summary of spatial information of Lv Liang sample with spatial coordinates, elevation and depths is also presented in Table 5. Table 6 summarises the basic physical properties of Lv Liang samples. Details of these can be found in the Supplementary Material Location Information.

**Table 5:** Location information for samples in Lv Liang summarised.

	Lontitude	Latitude	Elevation (m)	Depth (m)
Min	110$^\circ$ 30$^\prime {^\prime}$ 0$^\prime {^\prime}$	35$^\circ$ 42$^\prime$ 0$^\prime {^\prime}$	615	1.50
Q1	110$^\circ$ 48$^\prime$ 0$^\prime {^\prime}$	36$^\circ$ 16$^\prime$ 12$^\prime {^\prime}$	926	3.0
Median	111$^\circ$ 7$^\prime$ 44$^\prime {^\prime}$	36$^\circ$ 41$^\prime$ 24$^\prime {^\prime}$	1036	4.50
Mean	111$^\circ$ 6$^\prime$ 0$^\prime {^\prime}$	36$^\circ$ 54$^\prime$ 36$^\prime {^\prime}$	1040	6.48
Q3	111$^\circ$ 18$^\prime$ 0$^\prime {^\prime}$	37$^\circ$ 25$^\prime$ 48$^\prime {^\prime}$	1138	7.0
Max	112$^\circ$ 6$^\prime$ 0$^\prime {^\prime}$	38$^\circ$ 59$^\prime$ 24$^\prime {^\prime}$	1670	40.0

*Min* minimum, *Q1* 1st quantile, *Q3* 3rd quantile, *Max* maximum.

**Table 6:** Summary of basic physical properties for Loess samples; Lv Liang.

	Water	Dry density	Degree distribution
Min	2.800	1.350	0.2853
Q1	7.718	1.462	0.3718
Median	10.470	1.550	0.4010
Mean	10.555	1.571	0.4046
Q3	12.975	1.667	0.4375
Max	20.000	1.970	0.5419

*Min* minimum, *Q1* 1st quantile, *Q3* 3rd quantile, *Max* maximum.

### Indoor experiment

The collapsibility coefficients are obtained via in-lab experiments; the commonly employed one is the double oedometer method^55^. The trimmed sets are cut from the original undisturbed block samples. The Specification of Soil Test (SL237-1999)^54^ regularised the dry densities and water contents required for test samples. The dry densities are set to be 1.2, 1.3, 1.4, 1.5 and 1.6 referenced to the largest dry density of samples. The water contents are controlled to be $12\%$, $14\%$, $16\%$, $18\%$ and $20\%$. Eleven levels of pressures applied to samples are in sequence of 50, 100, 200, 300, 400, 600, 800, 1200, 1600 and 2000 in units of kPa. The samples are classified as stablised if the change is less than 0.1 mm per hour and water is added.

The coefficients of collapsibility are calculated for each sample with formula: $\delta _s = \frac{h - h'}{h_0},$ for *h* and $h'$ being the stablised height and the height after water added under the given pressure. $h_0$ refers to the initial height before pressure. The collapse potential of loess are often categorised into the following intervals: (0, 0.015], (0.015, 0.03], (0.03, 0.07] and (0.07, 1]. Each of these indicates non-collapsible, weakly collapsible, moderately collapsible and strongly collapsible respectively^56^.

The dry densities and water contents of samples are regularised under the Specification of Soil Test (SL237-1999)^54^. The dry densities are set to be 1.2, 1.3, 1.4, 1.5 and 1.6 referenced to the largest dry density of samples. The water contents are controlled to be $12\%$, $14\%$, $16\%$, $18\%$ and $20\%$. Eleven levels of pressures applied to samples are in sequence of 50, 100, 200, 300, 400, 600, 800, 1200, 1600 and 2000 in units of kPa.

**Figure 16:**
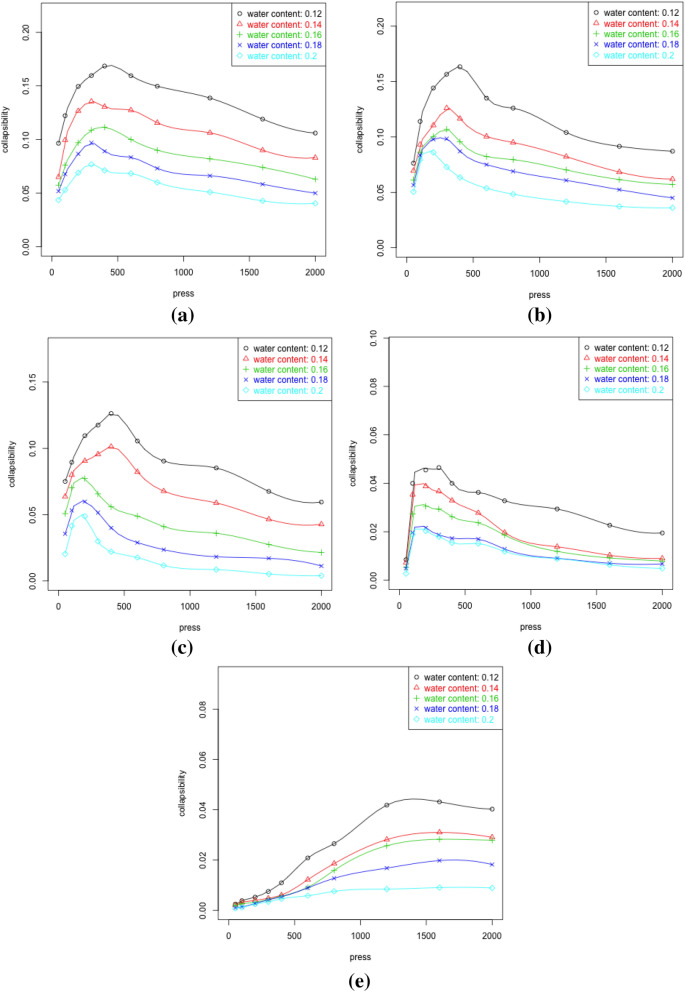
Variation of collapsibility corresponding to press; (**a**) dry density 1.2, (**b**) dry density 1.3, (**c**) dry density 1.4, (**d**) dry density, 1.5, (**e**) dry density1.6.

Upon the removal of initial states where pressure, water contents and deformation are all zeroes, collapsibility varies between 0.00085 and 0.16850 for all samples with different dry densities considered. The porosity ratio varies between approximately 0.41 and 1.06 with $75\%$ of values lying within the interval of 0.55 and 0.71. The press levels are controlled to take values between 0 and 2000 kpa for each different water contents within the five different samples corresponding to the dry density levels. Figure 16 shows the variation in collapsibility coefficients given different press levels for samples at give dry densities with different water contents. The clear differences at different levels of water contents can be seen for all samples. The patterns of peaking and steady states, however, are very similar at different water contents. In fact, regardless of dry densities, the lower the water contents, the higher overall collapsibility is shown. The collapsibility peaks with pressure reaching around 500 kpa and flattens off straight after for samples with dry densities of 1.2, 1.3, 1.4 and 1.5. With dry density of 1.6, the samples showed different behaviours as the increasing trend remained fairy steady with peak being reached around 1200 kpa.

### Statistical models

This paper employs a collection of modelling methods towards different targets. Multiple Linear modelling is adapted to establish the relationship between collapsibility and the in-lab physical properties of Yan’an samples. The predictor selection is based on the backward selection procedure with indication of AIC; the smaller the AIC value is, the more optimal the model is. This model is further validated and extended to the Lv Liang samples. As Lv Liang samples contain rich location information as to where the samples are taken from, a generalised additive model is used. The location information is smoothed by spline functions in this concept and thus extend to a 2-dimensional surface overlaid onto the Jin-Shan Loess Plateau. Details of these statistical methods are given in the Supplementary Material Model Details.

#### Generalised additive model

The generalised additive model is employed to take account of the spatial random effects both observed and evidenced above. The model details are omitted here but can be found in the Appendix. For *Y* denoting collapsibility coefficient, the spatial random effect model is written as:

$$\begin{aligned}{}& \log {Y_i} \sim {\mathcal {N}}(\mu _{i}, \sigma ^{2}_{i}), \\ & \mu _{i} = X_i\beta _i + S(X_{-i}) + S(x_i, y_i), \end{aligned}$$

where $X_i$’s are the fixed effects for sampling location *i*, $S(X_{-i})$ is the smoothed covariates (which have not been considered in $X_i$ and $S(x_i, y_i)$ refers to the smoothed spatial random effects. Thus, the generalised additive model the geographical locations with given coordinates (*x*, *y*) are taken into account as the smooth residual spatial variation. The porosity measurement contributes linearly towards the mean of log-collapsibility showing a positive direction. The basic physical properties including water contents and dry densities are included in as smoothed terms considering they are continuous numbers in nature. Similarly, the factors such as elevation and depth are also smoothed using cubic splines.

#### Model validation

**Figure 17:**
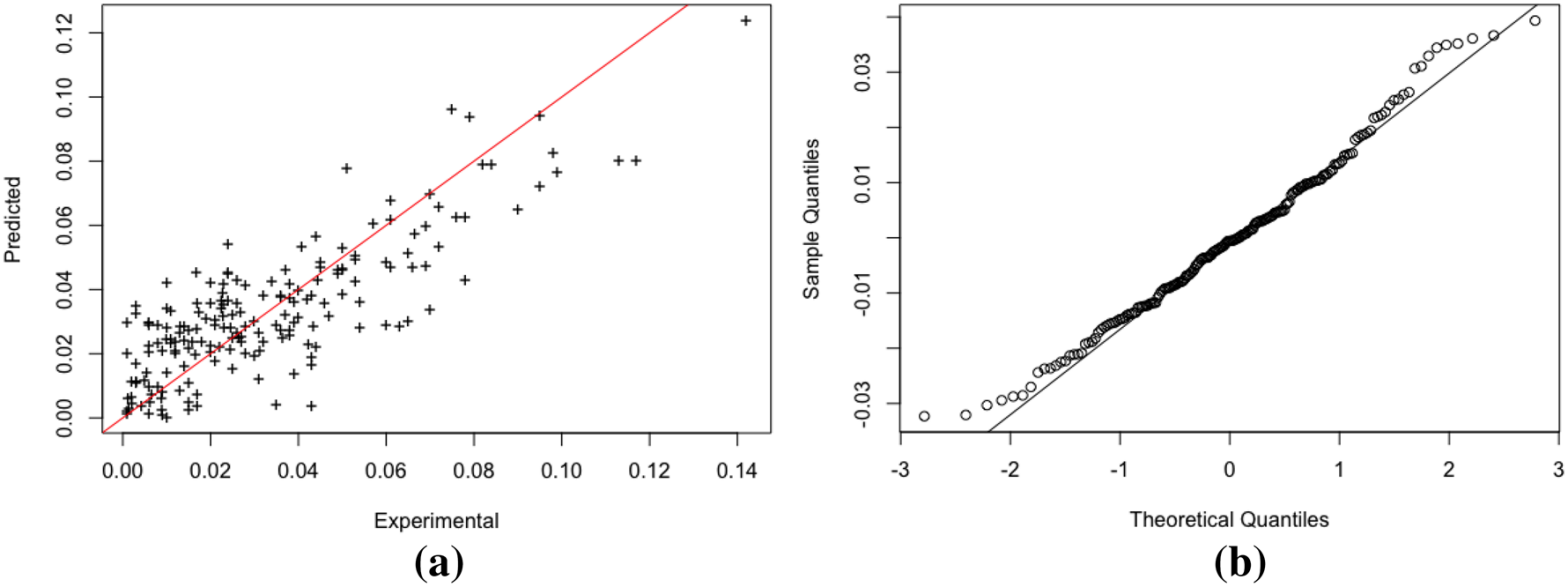
Generalised additive model validation: samples from Lv Liang; (**a**) Predicted vs experiment, (**b**) QQ-plot for residual checks.

The fitted generalised additive model explains $67\%$ of the deviance within the data. The only fixed effect term (porosity ratio) showed negative impact towards the log-transformed collapsibility coefficient. This is in line with the finding from previous research and the exploratory analysis. The model prediction is validated against the sample observed collapsibility values (Fig. 17a). The model residuals fall in line with the assumptions which supports that such choice of model is reasonable (Fig. 17b). Comparing to Figs. 3, 17 shows that predictions against observed are more evenly distributed along the line $y = x$; this means more agreement is seen between predicted and observed values. The QQ-plot also appeared to be in a better linear form indicating that residuals are more randomly distributed following a standard Normal distribution.

**Figure 18:**
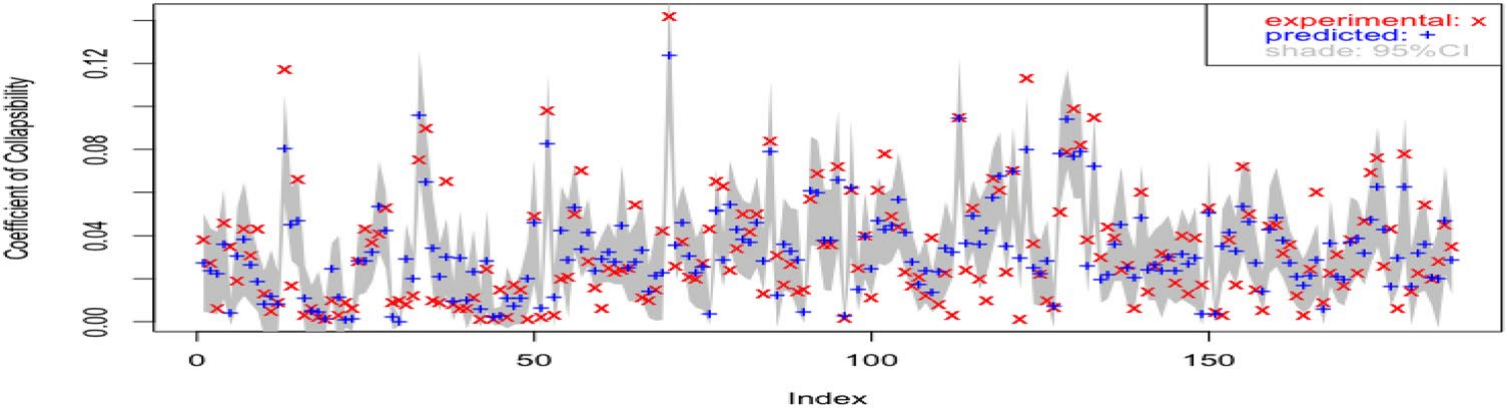
Generalised additive model predicted values with $95\%$ confidence interval overlaid by observed values: sample from Lv Liang.

Figure 18 shows that very rare ‘real’ values fall outside of the $95\%$ confidence interval. This is to say that the predicted values are rather robust and satisfactorily acceptable. Comparing to Fig. 3, a marginal improvement can be seen in predictions based on the generalised additive model incorporating spatial random effects.

## Supplementary Information

Supplementary Information 1.

Supplementary Information 2.

## Data Availability

All data used in the study are confidential in nature, so the research data are not shared.
