# Supplementary material for: A longitudinal analysis of collapsibility with predictions over the southeastern Loess Plateau in China
Source: Sci Rep. 2021 Dec 10;11:23824. doi: 10.1038/s41598-021-02623-2 (PMC8664886; doi:10.1038/s41598-021-02623-2)
Supplement: Supplementary file 1 — Supplementary Information 1. [file 41598_2021_2623_MOESM1_ESM.pdf]

APPENDIX

Multiple Linear Models

In the situation where the response variable is affect by a combination of factors, multiple regression models are often employed. For the response variable denoted y and covariates denoted x describes a model of the form $(y|X, \beta, \sigma) \sim \mathcal{N}(X\beta, \sigma^2 I_n)$, where β_1 is the intercept and β_j $(j \neq 0)$ is the slope corresponding to each observed covariate and e's are the identically and independently distributed error terms each following a Gaussian distribution $\mathcal{N}(0, \sigma^2)$. Here I_n is the identity matrix with diagonal entries being 1. Thus the probability density function for the response y has the form:

$$f(y|X, \beta, \sigma^2) = (2\pi\sigma^2)^{-n/2} \exp\{-\frac{1}{2\sigma^2}(y - X\beta)'(y - X\beta)\}.$$

Model estimates of responses and coefficients in multiple linear regression have the following forms of:

$$\hat{\beta} \sim \mathcal{N}(\beta, \sigma^2(X'X)^{-1})$$

$$\hat{y} \sim \mathcal{N}(X\beta, \sigma^2 H)$$

$$\hat{e} \sim \mathcal{N}(0, \sigma^2(I_n - H))$$

where $H = X(X'X)^{-1}X'$.

The coefficient of multiple determination is commonly used as an implication of how good

the model fit is. It takes values between 0 and 1 and indicates the amount of variation in y's that is explained by the model. However, the value of R^2 is sometimes artificially inflated when including more predictors. Thus throughout this paper, the adjusted R^2 will be reported as a relative measure of fit,

$$R^2_{\text{adjusted}} = 1 - \frac{\hat{\sigma}^2}{s_Y^2},$$

where $S_Y^2 = \frac{\sum_{i=1}^{n}(y_i - \bar{y})^2}{n-1}$ is the estimated sample variance.

Generalised Additive Models

Many data in reality do not follow a simple linear trend, thus allowing smoothing terms over the data is necessary to account for 'wiggly' shapes. [59] introduced a non-parametric extension which allow an additive functional form of covariates towards the linear restrictions in the model. This class of models is applicable to any random variables from the exponential family. A link function is introduced to draw relationship between covariates and expected responses. In formula, it can be written as

$$g(\mathbb{E}[y|u]) = \beta_0 + f_1(x_1) + \cdots + f_m(x_m) = \eta,$$

where f_i's are the smoothing functions and the expected value of $Y|u$ is related to covariates effects via its link function g. The generalised additive models capture non-linearities in the data via the smoothing functions f in forms of splines, polynomials or step functions etc and thus make inference about these smooth functions [52, 60, 61]. This allows an extra underlying random variation together with the Gaussian distribution. Random

effects are assumed to follow a Gaussian distribution with mean 0 and some unknown variance. They are combined with the fixed effects via linear predictor $\eta = X\beta + Zu$. This class of models is commonly used in clustered and hierarchical studies. For the purpose of this paper, it is employed to account for spatial autocorrelation by including a smoothed spatial location term over latitudes and longitudes of the data points [62]. On inference of the generalised additive models, each smooth function f can be represented as a sum of K fixed basis functions, eg. via B-spline methods [63].

Model Selection Procedures

The common model selection method normally aims to achieve the goal of obtaining the model with the best predictions. This does not imply that other models are 'incorrect'. The algorithm employed in this paper is the backward stepwise selection where the initial model is the full least squares model containing all predictors. One variable with the largest p-value (ie. least significant) is removed from the model iteratively for each step until the selection criteria is met. Here, the stop rule is set to be when the model appears to be the best in predictions. For this paper, the criterion employed to determine how good model fits are is Akaike Information Criterion (AIC) [64], defined to be $AIC(j) = 2n\hat{K}_j$ based on previous derivation and is used to indicate how good the model predictions are.
